# The prevalence of turnover intention and influencing factors among emergency physicians: a national observation

**DOI:** 10.1186/s12960-021-00688-8

**Published:** 2021-12-04

**Authors:** Shijiao Yan, Xin Shen, Rixing Wang, Zhiqian Luo, Xiaotong Han, Yong Gan, Chuanzhu Lv

**Affiliations:** 1 School of Public Health, Hainan Medical University, Haikou, Hainan China; 2 Key Laboratory of Emergency and Trauma of Ministry of Education, Hainan Medical University, Haikou, Hainan China; 3 Department of Social Medicine and Health Management, School of Public Health, Tongji Medical College, Huazhong University of Science and Technology, No. 13 Hangkong Road, Wuhan, 430030 Hubei China; 4 Department of Emergency, Hainan Clinical Research Center for Acute and Critical Diseases, The Second Affiliated Hospital of Hainan Medical University, Haikou, Hainan China; 5 Emergency and Trauma College, Hainan Medical University, Haikou, Hainan China; 6 Research Unit of Island Emergency Medicine, Chinese Academy of Medical Sciences (No. 2019RU013), Hainan Medical University, Haikou, Hainan China; 7 Department of Emergency Medicine, Hunan Provincial Institute of Emergency Medicine, Hunan Provincial Key Laboratory of Emergency and Critical Care Metabolomics, Hunan Provincial People’s Hospital/The First Affiliated Hospital, Hunan Normal University, Changsha, Hunan China; 8 Department of Emergency Medicine Center, Sichuan Provincial People’s Hospital, University of Electronic Science and Technology of China, Chengdu, China

**Keywords:** Turnover intention, Emergency medicine, Emergency physicians, Physicians shortage, Prevalence, China

## Abstract

**Background:**

Adverse consequences of physician turnover include financial losses, reduced patient satisfaction, and organizational instability. However, no study has reported the prevalence among emergency physicians. This study explore the rate and influencing factors of this community, which could provide a reference for preventing the loss of emergency physicians.

**Methods:**

A nationally representative cross-sectional survey of 15,243 emergency physicians was conducted in 31 provinces across China between July and September 2019. Multivariable logistic regression analysis was performed to identify predictors of turnover intention.

**Results:**

There were 49.75% of emergency physicians having turnover intention. Logistic regression analysis model showed that emergency physicians who were male (OR = 0.87) and older [> 37 and ≤ 43 (OR = 0.78) or > 43 (OR = 0.64)], worked in eastern China (OR = 0.88) and higher level of hospital [two-grade level (OR = 0.71) or three-grade level (OR = 0.56)], and had high (OR = 0.75) or middle (OR = 0.81) level income were not more likely to have less turnover intention, while those who had higher education level [bachelor degree (OR = 1.55) or master degree or higher (OR = 1.63)], long work tenure [> 3 and ≤ 6 (OR = 1.29) or > 6 and ≤ 11 (OR = 1.41) or > 11 (OR = 1.25)], poorer health status [fair (OR = 1.55) or poor (OR = 2.12)] and sleep quality [fair (OR = 1.16) or poor (OR = 1.43)], history of coronary heart disease (OR = 1.29), depression (OR = 2.77) and experienced the shift work (OR = 1.37) and workplace violence (OR = 1.78) were more likely to intend to leave.

**Conclusion:**

Nearly half of emergency physicians in China have turnover intention. Targeted intervening measures should be taken to reduce the turnover intention, so as to avoid the shortage of physicians and thus hinder the supply of emergency medical services.

## Background

Turnover is the actual leaving behavior essential to human resource management of a workforce [1]. Turnover intention refers to the psychological state before turnover, which indicates that the employee is not satisfied with the job, has the idea of resigning, looks for job opportunities, evaluates and compares other job opportunities. Intention to leave (turnover intention) is about an individual’s vision of a possible leaving and it often is studied as a proxy for actual turnover [2]. Previous studies have shown that work pressure, psychological problems and other factors have a significant impact on turnover intention [3, 4].

Physicians are one of the groups with high turnover intention and their turnover intention includes not only leaving the current post, but also transferring to other departments [5]. Previous systematic reviews have shown that about 3.2% to 53.7% of physicians have the turnover intention [5]. The generation of turnover intention is closely related to factors, such as work pressure and mental health [6–8], and its widespread prevalence will cause a shortage of doctors, and then produce diversified adverse results [9–12]. Specifically, the turnover of physicians threaten the operation of the health care system, may lead to the inadequate supply of health care services, thus impeding the masses' access to health care services, and even endangering the care and quality of life of patients [13–15]. In addition, adverse consequences of physician turnover include financial losses, reduced patient satisfaction, and organizational instability [16].

In providing medical services, emergency physicians are often faced with the problems of high work pressure, long shift time, large number of patients and various types of diseases; in the medical setting, they face staff shortages and the chaotic work environment characterized by unpredictability [17], especially in China. China receives the world's largest number of emergency patients each year (more than 166.5 million); China also has the largest number of emergency physicians, with nearly 60,000, accounting for more than 2.1% of the total number of physicians [18]. However, according to previous studies, emergency physicians in China face high work pressure and complex psychological problems [19], which laid a hidden trouble for the rise of turnover intention of emergency physicians [20].

Previous research on job stress among physicians has shown that emergency physicians have higher levels of stress (over 60%) compared to general physicians (38%) [21, 22]. However, it has not been reported whether emergency physicians have higher turnover intentions. Previous studies on turnover intention mostly focused on psychiatrists [23], general practitioners [24], emergency department nurses [25] and other groups. Therefore, the purpose of this study was to explore the prevalence of turnover intentions (including switching departments and turnover) of emergency physicians and influencing factors, which could provide a reference for global health-care policy makers and researchers to solve the shortage of physicians.

## Methods

### Ethics statement

The study protocol was approved by the Institutional Ethics Board of the Second Affiliated Hospital of Hainan Medical University, Haikou, China. All individuals provided written informed consent.

### Study participants and survey design

A cross-sectional study was carried out in China from July 2019 to September 2019. A multistage stratified random sampling design was used in this study. First, a total of 31 Chinese provinces were classified as developed, developing, or less-developed regions according to per capital household income in 2018. Second, we selected 10 hospitals randomly from each province. Third, based on the number and scale of the hospitals, 40% of the emergency physicians who had practiced in the emergency department for at least 6 months were randomly selected from each hospital to complete a self-administered questionnaire. The participants included only emergency physicians and not nurses and other technicians who provided emergency care. In total, 15,455 emergency physicians were asked to participate in this survey, and 182 physicians did not respond. In addition, 30 questionnaires were discarded due to missing information. Ultimately, 15,243 eligible questionnaires were used in this analysis.

### Instrument and measurement

The questionnaire was designed based on literature reviews, group discussions, and preliminary interviews. Furthermore, a pilot study was conducted in one Wuhan community to improve the quality of the questionnaire. This questionnaire focuses on the physical and mental health and professional attitude of emergency physicians, and the relevant parts of this study include sociodemographic information (e.g., region, age, gender, education level, marital status, and professional title), workplace violence, depression and turnover intention. The “turnover intention” section have one items, which is “Wether you have turnover intention”. The answers can be either "yes" or "no".

### The Chinese version of the Workplace Violence Scale (WVS)

WVS was developed by Wang et al. [26], has a good reliability and validity for measuring the incidence of workplace violence when applied to medical staff in China. It includes 5 items measured with a four-point ordinal scale ranging from 0 (never) to 3 (more than 3 times/year). In this study, the Cronbach’s alpha for WPV was 0.81.

### The Center for Epidemiological Studies Depression scale (CES-D)

CES-D was used to assess depressive symptoms. The scale includes include 20 items; each item is scored on a four-point scale ranging from 0 ("little or none of the time") to 3 ("most or almost all of the time"). The total score ranges from 0 to 60 points, and the higher the score is, the more severe the depressive symptoms. On the original CES-D scale, a total score of 16 was used to detect the presence of depressive symptoms [27]. However, a large number of studies have assessed the diagnostic accuracy of CES-D in detecting depression in the general population and have proposed multiple cutoff points, such as a cutoff point of 18 for elderly people living in residential homes [28] and a cutoff score of 22 for older Chinese individuals [29]. A meta-analysis systematically reviewed 28 CES-D studies, including several Chinese studies, and obtained an optimal cutoff point of 20 points [30]. As a result, an overall score of 20 or higher was considered an indicator of depressive symptoms in this study, consistent with previous research [31]. The CES-D has good reliability and validity, and it has been widely used in the Chinese population. In this study, the Cronbach's alpha coefficient of the scale was 0.90.

### Data collection and quality control

A web link to the online questionnaire, which was designed using Questionnaire Star, was disseminated to the participants through WeChat (similar to WhatsApp, WeChat is the largest communication platform in China, with over one billion users). To prevent the same participants from repeatedly answering the questionnaire, each device (e.g., smartphone or computer) was eligible to complete the questionnaire only once, and logical checks were concurrently run on the WeChat platform to identify invalid questionnaires. The data were entered into a web-based database by trained investigators to ensure accuracy.

### Statistical methods

The descriptive analyses used means for the continuous variables and percentages for the categorical data. This study examines the effects of both single and multiple factors. The descriptive statistics for the categorical variables were reported as the frequency (percentage) and were compared between “yes” and “no” using Pearson’s *χ*^*2*^ test. Weather having turnover intention as the dependent variables, were treated as categorical variables. Multivariable logistic analysis was used to calculate the odds ratios (ORs) and 95% confidence intervals (CIs) for factors that might be associated with the prevalence of turnover intention. All analyses were performed using STATA 13.0, and all tests were two sided with a significance level of 0.05.

## Results

Table 1 presents the main characteristics of the survey respondents. Among the 15,243 respondents, most were married (83.26%), were men (69.87%) and had a bachelor’s degree or above (94.18%). The mean age (SD) was 37.66 (8.06) years. Approximately 65% of the participants had intermediate or senior professional titles. In total, 55.23% of the respondents were in the middle-income and lower income groups, more than half estimated their own health to be at the general level, and approximately 40% were currently employed in eastern China. A minority of the participants (27.19%) participated in regular physical activity. 89.87% of respondents reported exposure to WPV. In addition, 7584 (49.75%) of emergency physicians had turnover intention. (Table 1).

**Table 1:** Statistical description of study samples

Variables	*N* (%)	Having turnover intention	*χ*^*2*^	*P* value
Total	15,243 (100.00)	7584 (49.75)	NA	NA
Gender				
Male	10,650 (69.87)	5443 (51.11)	25.92	** < 0.01**
Female	4593 (30.13)	2141 (46.61)		
Age group, y				
≤ 31	4089 (26.83)	1993 (48.74)	168.23	** < 0.01**
> 31 and ≤ 37	4117 (27.01)	2309 (56.08)		
> 37 and ≤ 43	3291 (21.59)	1714 (52.08)		
> 43	3746 (24.58)	1568 (41.86)		
Region				
Eastern China	6000 (39.36)	2843 (47.38)	22.25	** < 0.01**
Central China	4097 (26.88)	2102 (51.31)		
Western China	5146 (33.76)	2639 (51.28)		
Education level				
Associate’s degree or vocational diploma	894 (5.86)	343 (38.37)	53.39	** < 0.01**
Bachelor degree	10,293 (67.53)	5249 (51.00)		
Master degree or higher	4056 (26.61)	1992 (49.11)		
Marital status				
Married/widow/divorced	12,691 (83.26)	6342 (49.97)	1.45	0.23
Unmarried	2552 (16.74)	1242 (48.67)		
Income status				
High	1672 (10.97)	655 (39.17)	202.06	** < 0.01**
Middle	6746 (44.26)	3128 (46.37)		
Low	6825 (44.77)	3801 (55.69)		
Work tenure, y				
≤ 3	4921 (32.28)	2249 (45.70)	124.93	** < 0.01**
> 3 and ≤ 6	3114 (20.43)	1685 (54.11)		
> 6 and ≤ 11	3424 (22.46)	1907 (55.70)		
> 11	3784 (24.82)	1743 (46.06)		
Contract status				
Permanent	9715 (63.75)	4802 (49.43)	1.13	0.29
Temporary	5528 (36.27)	2782 (50.33)		
Professional title				
Elementary or below	5349 (35.09)	2619 (48.96)	88.22	** < 0.01**
Intermediate	5861 (38.45)	3168 (54.05)		
Senior	4033 (26.46)	1797 (44.56)		
Ownership				
Governmental	14,599 (95.78)	7272 (49.81)	0.46	0.50
Non-governmental	644 (4.22)	312 (48.45)		
Level of hospital				
Three-grade level	10,152 (66.60)	4921 (48.47)	20.07	** < 0.01**
Two-grade level	4841 (31.76)	2535 (52.37)		
Other	250 (1.64)	128 (51.20)		
Shift work				
Yes	13,288 (87.17)	6918 (52.06)	220.77	** < 0.01**
No	1955 (12.83)	666 (34.07)		
Workplace violence				
Yes	13,699 (89.87)	7171 (52.35)	363.71	** < 0.01**
No	1544 (10.13)	413 (26.75)		
Self-perceived health status				
Good	4707 (30.88)	1581 (33.59)	961.79	** < 0.01**
Fair	7729 (50.71)	4045 (52.34)		
Poor	2807 (18.42)	1958 (69.75)		
BMI (kg/m^2^)				
< 25	9588 (62.90)	4735 (49.38)	1.41	0.24
≥ 25	5655 (37.10)	2849 (50.38)		
History of hypertension				
Yes	2317 (15.20)	1274 (54.98)	29.91	** < 0.01**
No	12,926 (84.80)	6310 (48.82)		
History of diabetes				
Yes	598 (3.92)	308 (51.51)	0.76	0.38
No	14,645 (96.08)	7276 (49.68)		
History of coronary heart disease				
Yes	471 (3.09)	308 (65.39)	47.55	** < 0.01**
No	14,772 (96.91)	7276 (49.26)		
Smoking status				
Nonsmokers	12,287 (80.61)	6000 (48.83)	21.54	** < 0.01**
Smokers	2956 (19.39)	1584 (53.59)		
Alcohol drinking				
Nondrinkers	11,451 (75.12)	5630 (49.17)	6.37	**0.01**
Drinkers	3792 (24.88)	1954 (51.53)		
Physical inactivity				
Yes	11,098 (72.81)	5737 (51.69)	61.44	** < 0.01**
No	4145 (27.19)	1847 (44.56)		
Sleep quality				
Good	2295 (15.06)	735 (32.03)	704.34	** < 0.01**
Fair	7347 (48.20)	3348 (45.57)		
Poor	5601 (36.74)	3501 (62.51)		
CES-D scores				
< 20	9818 (64.41)	3735 (38.04)	1513.55	** < 0.01**
≥ 20	5425 (35.59)	3849 (70.95)		

The factors associated with the emergency physicians having turnover intention are presented in Table 2. Emergency physicians who were male (OR = 0.87) and older [> 37 and ≤ 43 (OR = 0.78) or > 43 (OR = 0.64)], worked in eastern China (OR = 0.88) and higher level of hospital [two-grade level (OR = 0.71) or three-grade level (OR = 0.56)], and had high (OR = 0.75) or middle (OR = 0.81) level income were not more likely to have less turnover intention, while those who had higher education level [bachelor degree (OR = 1.55) or master degree or higher (OR = 1.63)], long work tenure [> 3 and ≤ 6 (OR = 1.29) or > 6 and ≤ 11 (OR = 1.41) or > 11 (OR = 1.25)], poorer health status [fair (OR = 1.55) or poor (OR = 2.12)] and sleep quality [fair (OR = 1.16) or poor (OR = 1.43)], history of coronary heart disease (OR = 1.29), depression (OR = 2.77) and experienced the shift work (OR = 1.37) and workplace violence (OR = 1.78) were more likely to intend to leave.

**Table 2:** Logistic regression analysis on the influencing factors of Emergency physicians’ intentions to turnover in China

Variables	Coefficient	*S.E*	*P*	*OR*	95% *CI*
Gender (Ref: Female)					
Male	− 0.14	0.04	**0.01**	0.87	0.80–0.94
Age group, y (Ref: ≤ 31)					
> 31 and ≤ 37	− 0.05	0.06	0.47	0.96	0.85–1.08
> 37 and ≤ 43	− 0.25	0.08	**0.01**	0.78	0.67–0.91
> 43	− 0.45	0.09	** < 0.01**	0.64	0.54–0.76
Region (Ref: Western China)					
Eastern China	− 0.13	0.04	**0.03**	0.88	0.81–0.96
Central China	− 0.02	0.05	0.72	0.98	0.90–1.08
Education level (Ref: Associate’s degree or vocational diploma)					
Bachelor degree	0.44	0.08	** < 0.01**	1.55	1.32–1.82
Master degree or higher	0.49	0.09	** < 0.01**	1.63	1.36–1.95
Marital status (Ref: Married/widow/divorced)					
Unmarried	0.04	0.06	0.43	1.04	0.94–1.16
Income level (Ref: Low)					
High	− 0.29	0.06	** < 0.01**	0.75	0.66–0.85
Middle	− 0.21	0.04	** < 0.01**	0.81	0.75–0.87
Work tenure, y (Ref: ≤ 3)					
> 3 and ≤ 6	0.25	0.05	** < 0.01**	1.29	1.16–1.43
> 6 and ≤ 11	0.34	0.06	** < 0.01**	1.41	1.26–1.58
> 11	0.22	0.06	**0.01**	1.25	1.10–1.41
Contract status (Ref: Temporary)					
Permanent	0.01	0.04	0.75	1.01	0.93–1.10
Professional title (Ref: Elementary or below)					
Intermediate	0.14	0.06	**0.02**	1.15	1.03–1.29
Senior	0.29	0.08	** < 0.01**	1.34	1.14–1.56
Ownership (Ref: Non-governmental)					
Governmental	− 0.13	0.09	0.15	0.88	0.74–1.05
Level of hospital (Ref: Other)					
Three-grade level	− 0.58	0.14	** < 0.01**	0.56	0.43–0.74
Two-grade level	− 0.34	0.14	**0.02**	0.71	0.54–0.94
Shift work (Ref: No)					
Yes	0.32	0.06	** < 0.001**	1.37	1.22–1.55
Workplace violence (Ref: No)					
Yes	0.58	0.06	** < 0.01**	1.78	1.57–2.02
Self-perceived health status (Ref: Good)					
Fair	0.43	0.04	** < 0.01**	1.55	1.42–1.68
Poor	0.75	0.06	** < 0.01**	2.12	1.88–2.39
BMI (kg/m^2^) (Ref: < 25)					
≥ 25	− 0.07	0.04	0.09	0.94	0.87–1.01
History of hypertension (Ref: No)					
Yes	0.04	0.05	0.52	1.04	0.93–1.15
History of diabetes (Ref: No)					
Yes	− 0.12	0.10	0.22	0.89	0.74–1.07
History of coronary heart disease (Ref: No)					
Yes	0.27	0.11	**0.02**	1.29	1.04–1.61
Smoking status (Ref: Nonsmokers)					
Smokers	0.05	0.05	0.27	1.06	0.96–1.16
Alcohol drinking (Ref: Nondrinkers)					
Drinkers	0.08	0.05	0.09	1.08	0.99–1.18
Physical inactivity (Ref: No)					
Yes	− 0.05	0.04	0.24	0.95	0.88–1.03
Sleep quality (Ref: Good)					
Fair	0.15	0.06	**0.01**	1.16	1.04–1.29
Poor	0.36	0.06	** < 0.01**	1.43	1.27–1.61
CES-D Scores (Ref: < 20)					
≥ 20	1.02	0.04	** < 0.01**	2.77	2.56–3.00

## Discussion

To our knowledge, this is the largest survey of physicians' turnover intentions in the world, and we focus on emergency physicians, a community of physicians with high levels of work stress. We found that nearly half (49.75%) of emergency physicians had turnover intention. Compared to previous studies of psychiatrists (20%) [23], general practitioners (47%) [24], and emergency department nurses (23%) [25], emergency physicians showed higher levels of turnover intention, which indicated that fully attention should be paid to this community. In addition, we identified a number of representative factors that clearly correlate with the increasing of turnover intention among emergency physicians, which could provide references for targeted measures.

### Age, gender, region, level of hospital and income level

Our study found that emergency physicians who were older, male, worked in eastern China, and had high income levels were less likely to develop turnover intention. These findings are consistent with previous studies. Eastern China is economically developed, while central China and western China are relatively backward. Lu et al. [32] found that that physicians who are younger and work in under-development region had higher turnover intention because of low salaries and lack of promotion prospect. The same phenomenon also could happen in lower level hospitals. While Sperlich et al. [33] reported that women are more likely to be influenced by family and social factors at work and have higher levels of work stress which is an important reason for their turnover intention [34]. In addition, some studies mention that attractive salary is the pivotal factor to increased job satisfaction and reduce turnover intention [35, 36]. Low salary can make it difficult to attract and retain new employees. Fang et al. [37] also found that the lower the salary level, the more likely physicians were to develop turnover intention. This suggests that more attention should be given to emergency doctors who are younger, female, work in less developed areas and lower level hospitals, and have poor salary.

### Education level, professional title and work tenure

Previous research has shown that well-educated workers are more likely to leave for career development [38, 39]. This situation is further aggravated if career opportunities within the organization are limited [40]. We also found that highly educated emergency physicians were more likely to have turnover intention. In addition, the sense of achievement is also an important factor that affects work enthusiasm [41]. Emergency physicians with high professional titles and longer working tenure may experience a decreased sense of professional accomplishment and turnover intention. In addition, longer working tenure may lead to fatigue, tension and burnout, which has an indirect effect on the turnover intention [42]. This is consistent with our findings and indicated that the turnover intention of emergency physicians with high professional titles and long working experience should not be ignored. In reality, these community are often responsible for crucial medical serves.

### Health status, sleep quality, and depression

Emergency physicians bear high physical and psychological job demands that may jeopardize their health status. Illness and health problems are the main causes of turnover intention [43]. Our study showed that emergency physicians with poor physical health were more likely to report turnover intention. In addition, the history of coronary heart disease is also significantly related to turnover intention. The discovery is the first reported and needs further exploration. The decline in sleep quality reflects that physicians may have higher work pressure, which is ultimately translated into turnover intention [34]. Heponiemi et al. showed a positive association between decreased sleep quality and physicians' intentions to change their profession [44]. The decline in sleep quality could lead to mood disorders, such as depression [45, 46], both of which lead to chronic fatigue. Moreover, depression may decrease concentration, which reduces the productivity of physicians and affects physicians’ judgment, thus increasing occupational injury and turnover intention [47, 48]. Therefore, the managers of health institutions should commence to improve the physical health of emergency physicians, and avoid their psychological problems, which could decline their turnover intention.

### Shift work and workplace violence

Some studies have found that shift work is associated with an increase in turnover intention; moreover, reducing restrictions on working hours and providing more flexible shift work will have a positive impact on reducing turnover intention [49–51]. Our study also demonstrates that shift work increases the turnover intention among emergency physicians. On the other hand, workplace violence in emergency department has received increasing attention in recent years [52, 53]. The survey of Occupational Health Safety Network (OHSN) in America during 2012–2015 showed the incidence of emergency room violence was 19.3%, with an average annual growth rate of 23% [54]; and previous studies have shown that only 30% of cases were documented [55, 56], while nearly 80% of cases was undocumented [57]. Heponiemi et al. [58] reported workplace violence in the health care sector may lead to increased turnover and job dissatisfaction, which is consistent with our findings. Therefore, targeted measures are needed to optimize the health service system, provide more flexible working regimes for emergency physicians, and mitigate doctor–patient relationships to reduce workplace violence.

It is worth noting that among the factors identified, some are "push" factors (e.g., violence, poor health and shift work) and others are "pull" factors (e.g., ambition and career development). Comprehensive measures could reduce turnover intention more effectively. In the process of developing targeted measures, the government and medical institutions should pay more attention to eliminating the negative impact of "push" factors and improving the retention rate. Moreover, for the "pull" factor, it is important to provide sufficient development opportunities for physicians. Proper turnover is acceptable.

The shortage of physicians is the focus of the current medical service field, but there is no effective solution to this problem. It might be a potential direction to commence by reducing turnover intention. This study is based in China, the largest developing country. Currently, many countries have the similar incomplete medical service system, and are also faced with excessive medical demand. This contradiction is particularly evident in the global COVID-19 pandemic and also creates a higher workload for physicians, especially emergency physicians. Therefore, researching the prevalence and influencing factors of turnover intention of emergency physicians in China will provide a valuable reference for researchers and policy makers of the global emergency health service system.

### Strengths, limitations and future suggestions

Our study used a large-sample cross-sectional survey to explore the influencing factors of turnover intention among emergency physicians. First, the large sample size significantly increased the statistical power. We surveyed nearly a quarter of the nation's emergency physicians, so the participants was representative. Second, this study included more than twenty potential contributors and produced many valuable findings, which reflects their working status and presented a broad view of the challenges faced by emergency physicians. Third, the survey was anonymous and self-administered, which likely made the respondents provide more valid responses and eliminated interviewer bias.

This study had several limitations. First, the study used a cross-sectional study design, which precluded the evaluation of the temporality of the observed relationships. Second, the data were collected from the participants’ self-reports; thus, recall bias was unavoidable. Finally, the study was performed before the COVID-19 pandemic, which may have led to changes in the turnover intention of emergency physicians after the pandemic.

Based on our findings, we suggest that, first, prospective studies are needed to investigate the association between the identified factors and turnover intention. Further research should also conducted into the post-turnover whereabouts of emergency physicians. Second, this study highlights the need for the investigation or implementation of interventions to improve emergency physicians’ well-being or promote strategies to reduce work pressure on healthcare settings. Finally, investigating the potential impact of turnover intention on emergency physicians’ work performance, the quality of patient care delivery, and family life would provide important insights.

## Conclusion

Our study found that nearly half of emergency physicians in China have turnover intention which is influenced by a variety of factors. Targeted intervening measures should be taken to reduce the turnover intention, so as to avoid the shortage of physicians and thus hinder the supply of emergency medical services.

## Data Availability

Data may be made available by contacting the corresponding author.

## Abbreviations

BMI: Body Mass Index
CES-D: Center for Epidemiological Studies-Depression Scale
